# The Jenner Institute of Preventive Medicine

**Published:** 1903-07-11

**Authors:** 


### The Jenner Institute of Preventive Medicine.

The antitoxin treatment of disease has attained vast dimensions in every civilised country, although the doctrine of immunity upon which it is based is only of recent origin. Too much at first was expected of the antitoxin theory, because it was hoped that it would prove to be curative. But experience has taught it is only prophylactic, and that to be of the greatest service it must be adopted soon after the patient has been infected and before the serious symptoms have been produced. The antitoxin theory of the treatment of diphtheria and tetanus has established itself on a firm basis, and there are probably very few cases of either disease in which the patients are not subjected to one or more injections. The demand for these sera is therefore great. Typhoid inoculations and injections of cultures of streptococci and dysentery are coming into use, and there are several others which as yet have only reached the experimental stage. The serum in each case can only be produced by very skilled labour, for it involves constant attention to the most minute detail. Its production must necessarily be carried out with great care, for disastrous accidents on a large scale have resulted from the accidental contamination of the serum employed for one disease with that prepared for another. Above all, it is necessary to standardise the strength of the serum in each case, because the method of obtaining antitoxins of high value, though apparently based on sound theoretical grounds, is found often to be practically defective.

The Jenner Institute of Preventive Medicine has always done excellent work in connection with the manufacture and distribution of antitoxic sera in their most reliable forms, and has maintained a large department for the supply of serum. The department used to be situated at Sudbury, near Harrow, but in consequence of the compulsory disturbance to make room for the Great Central Railway, it became necessary to find a new site. The Institute was fortunate in securing a breeding stable, with first-rate accommodation, for 36 horses, a little distance from Elstree, which is only 11 miles from London, straight up the Edgware Road. The buildings stand on a site of about 28 acres of grass land, and are surrounded on all sides by open fields. Dr. Dean, the bacteriologist in charge, lives in a separate house, whilst the assistant bacteriologists, Dr. Todd and Dr. Petrie, occupy the original building known as Queensberry Lodge. A very complete suite of laboratories has been built by the Institute from designs by Mr. Paul Waterhouse. The laboratories are constructed, according to the latest requirements, with papyrolith floors; they have rounded cornices, glazed adamant walls, white tiles, and large windows, with red glass where necessary to shut off the actinic rays of light. The laboratories consist of a room where the ordinary work of the establishment is conducted, of two private laboratories for original research, and of a laboratory used entirely for filtering and storing the different varieties of serum. Every precaution is taken to ensure the purity of the serum. It is separated from the clot with aseptic precautions, and is filtered through Berkfeld filters into the large flasks in which it is stored. The flasks connected with the filters are sterilised just before they are filled, and the small phials in which the serum is sent out for use are filled from them. The bottling-room where the small phials are filled is air-tight and is washed out and disinfected with formalin vapour, which is allowed to remain in the room all night. The vapour is expelled by a current of air, sterilised by passing through a large cotton-wool filter occupying one side of the room near the floor, an exit plugged with wool being provided in the floor at the opposite side. The floor is then swabbed with oil to prevent any dust rising, and the current of air is maintained whilst this is being done. A positive air pressure is thus obtained, so that when the door is opened the air flows outwards only, and a practically sterile atmosphere can be obtained. The actual filling of the phials with serum has always been done personally by one of the staff. Finally, an isolated laboratory stands entirely apart from any other building, for the preparation and filtration of tetanus toxin. It serves too as a post-mortem room for small animals. The greenhouses of Queensberry Lodge are retained and make admirable animal houses for the rabbits, guinea-pigs, and monkeys.

Lord Lister and the governing body of the Jenner Institute of Preventive Medicine invited a large company to inspect the laboratories and stables on Friday, July 3rd. The invitation was very generally accepted, and many distinguished members of the public health service, with representatives of the pathological, medical, and surgical sides of the profession, assembled at St. Pancras station at 2.15 P.M. on that day. They were conveyed by special train to Elstree, where wagonettes carried them to Queensberry Lodge, situated some three miles from the station. The party was conducted over the buildings by Dr. Dean, Dr. Todd, and Dr. Petrie. The visitors inspected the whole establishment with the interest of experts, and afterwards expressed themselves greatly pleased with the construction and arrangement of the premises. Tea was served in a marquee on the lawn, and London was again reached at seven o'clock in the evening.

It seems probable that the Jenner Institute of Preventive Medicine will soon return to its former title of the British Institute of Preventive Medicine, owing to the difficulty which has arisen in connection with the manufacture of calf lymph on premises situated near those occupied by the Institute. The manufacturer of the lymph calls his factory the "Jenner Institute for Calf Lymph," and was using Jenner's name, says our contemporary the *Lancet*, as a hall-mark to his wares before the British Institute of Preventive Medicine altered its title. The Jenner Institute of Preventive Medicine accordingly entered into an agreement not to manufacture calf lymph, as it seemed impossible to do so without causing confusion in the public mind, whilst an attempt to purchase the manufacturer's prior right ended in failure. The Jenner Institute has now determined to alter its name, in order to produce calf lymph which shall have no chance of being confounded with that produced by the private manufacturer.

